# Molecular biology approaches in bioadhesion research

**DOI:** 10.3762/bjnano.5.112

**Published:** 2014-07-08

**Authors:** Marcelo Rodrigues, Birgit Lengerer, Thomas Ostermann, Peter Ladurner

**Affiliations:** 1 University of Innsbruck, Institute of Zoology and Center for Molecular Biosciences Innsbruck, Technikerstraße 25, A-6020 Innsbruck, Austria

**Keywords:** bioadhesion, differential gene expression, in situ hybridization, RNA interference, transcriptome

## Abstract

The use of molecular biology tools in the field of bioadhesion is still in its infancy. For new research groups who are considering taking a molecular approach, the techniques presented here are essential to unravelling the sequence of a gene, its expression and its biological function. Here we provide an outline for addressing adhesion-related genes in diverse organisms. We show how to gradually narrow down the number of candidate transcripts that are involved in adhesion by (1) generating a transcriptome and a differentially expressed cDNA list enriched for adhesion-related transcripts, (2) setting up a BLAST search facility, (3) perform an in situ hybridization screen, and (4) functional analyses of selected genes by using RNA interference knock-down. Furthermore, latest developments in genome-editing are presented as new tools to study gene function. By using this iterative multi-technologies approach, the identification, isolation, expression and function of adhesion-related genes can be studied in most organisms. These tools will improve our understanding of the diversity of molecules used for adhesion in different organisms and these findings will help to develop innovative bio-inspired adhesives.

## Introduction

**Figure 1:**
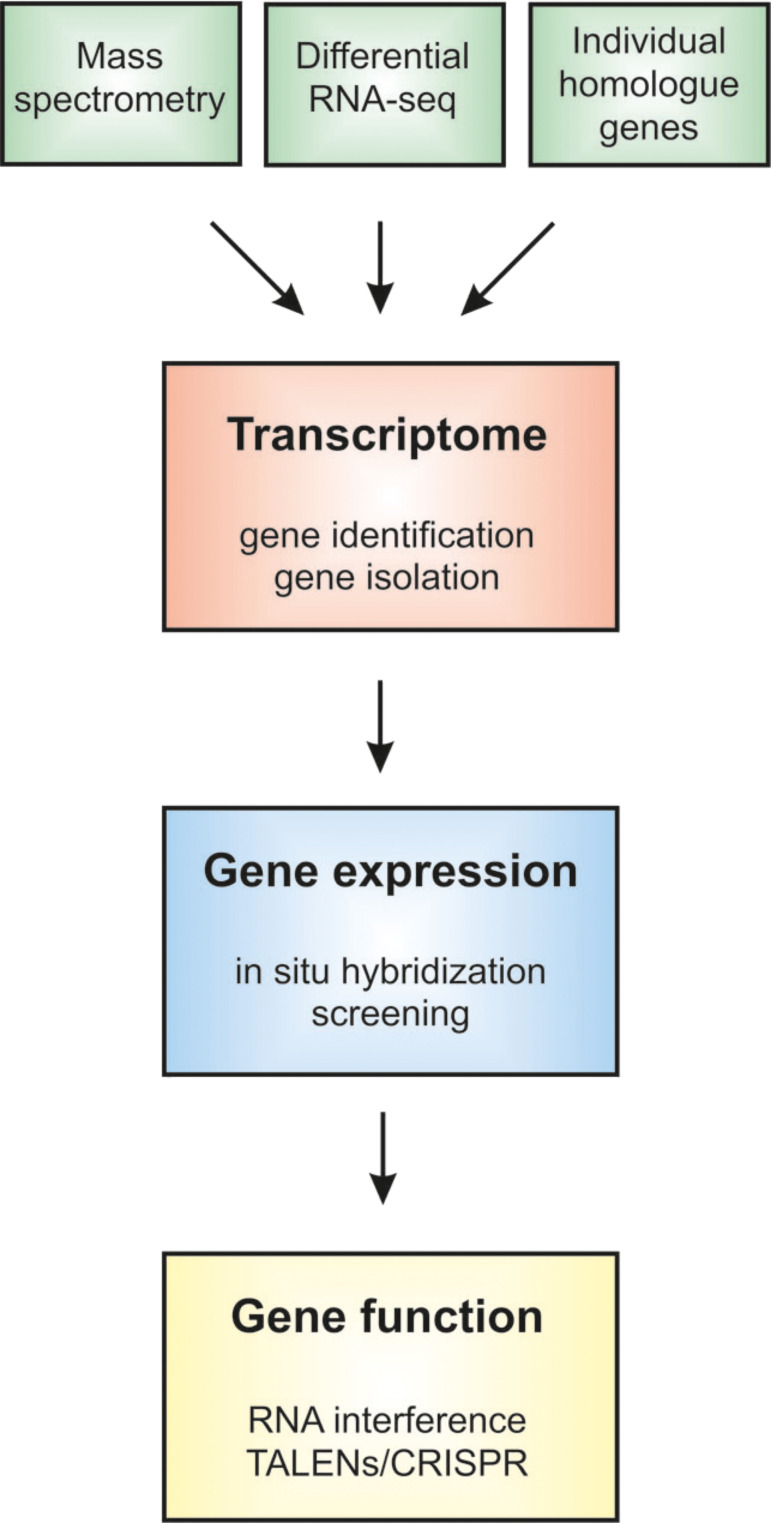
Flowchart showing the transcriptome as a central element to be searched by data generated from various sources, while downstream analyses also rely on the transcriptome.

The capability of an organism to attach to a surface, either temporarily or permanently, is referred to as “bioadhesion”. Bioadhesion occurs in many living organisms that have designed ways to adhere to a range of surfaces [1–3]. Information on how animals solve problems of adhesion in diverse environments can lead to the development of novel bio-inspired adhesives [4] with major applicability in the fields of surface engineering and biomedicine. Molecular biology is helpful in bioadhesion research with respect to the isolation of genes, and the study of their expression and function (Figure 1). Methods in the field have advanced tremendously in recent years, largely due to the recent advances in DNA and RNA sequencing, and protein analysis. These technologies allow research objectives to move from the analyses of single genes to the study of more complete sets of genes, or to examine all genes that are expressed at once. Now, functional genomics may reveal the transcriptional program of entire genomes by RNA sequencing.

The recent advances in molecular biology have made available a wide range of research tools and techniques that are of particular interest to researchers working on bioadhesion of organisms where no reference genome exists. Important prerequisites of bioadhesion research are based on techniques such as histology, biochemistry and mechanics [1,3] but gradually certain model systems are entering molecular biology such as mussels [5], barnacles [6–7], sandcastle worms [8], starfishes [9], and flatworms [10]. Efforts to develop bio-inspired adhesives are most effective when guided by a detailed understanding of the key features and mechanisms of natural adhesives [11]. Here, we intend to provide a general outline of cutting-edge methods in molecular biology from which researchers can explore the mechanism of biological adhesion. We know that no single protocol can be applied for every organism. Our goal is to offer a conceptual design of molecular biology tools for experimental analysis ranging from gene identification to gene function in bioadhesion.

The article is divided into three main sections: firstly, we describe the generation of a transcriptome and the use of the differential transcriptome in order to attain the full complement of transcripts (we refer to as bioinformatically assembled hypothetical complementary DNA originating from isolated messenger RNA) expressed in the region of the animal containing adhesive-producing cells; secondly, in situ hybridization (ISH) screening provides the (temporal and) spatial expression of target transcripts; thirdly, RNA interference (RNAi) allows for the elucidation of selected genes by their manipulation in vivo. These tools provide highly detailed molecular information about the adhesive-related proteins. This would impact mainly research on permanent adhesives made up of a combination of carbohydrates and proteins. Indeed, even temporary adhesives that contain a significant carbohydrate fraction usually also rely on proteins for adhesion.

## Review

### 1. Transcriptome sequencing and differential gene expression

#### 1.1 What is a transcriptome?

**Figure 2:**
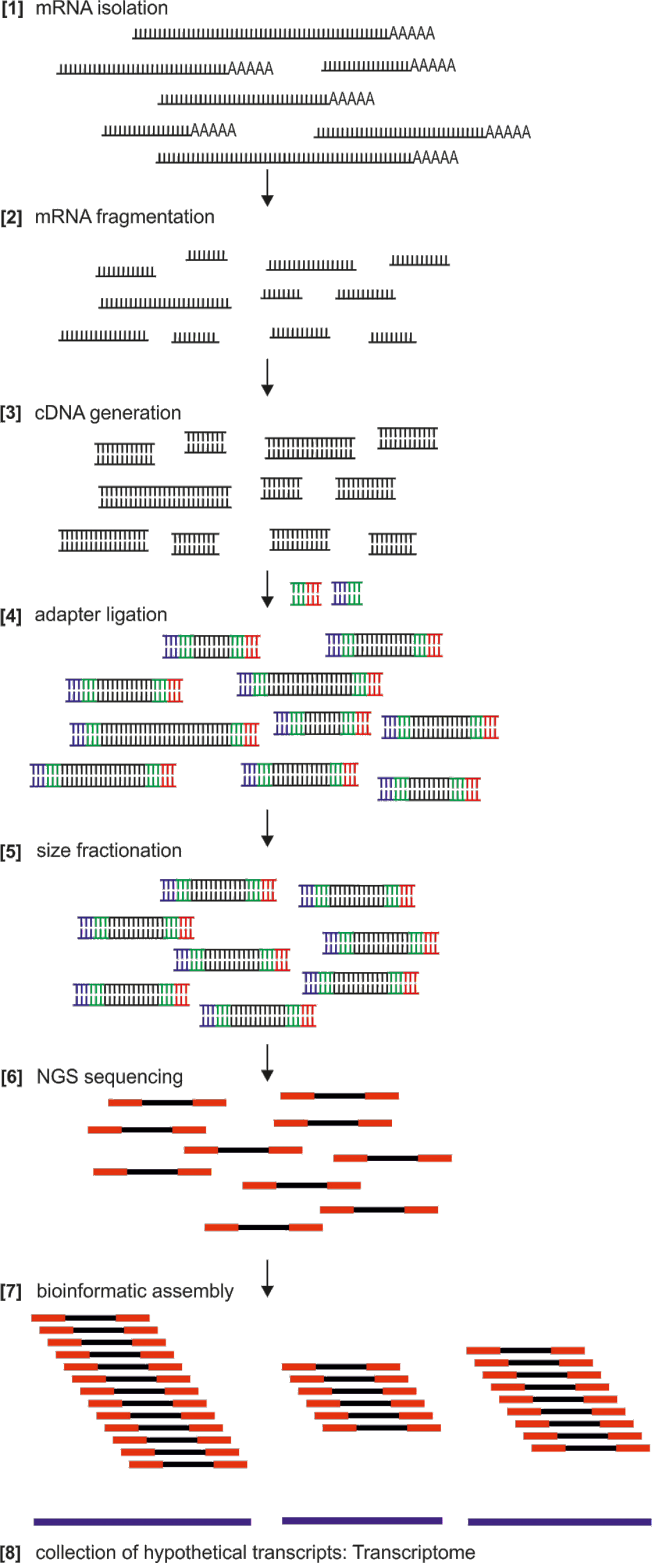
Next generation sequencing (NGS) is a massive sequencing technology, which enables hundreds of gigabases of data to be produced in a single sequencing run. Here, the consecutive steps for the generation of a transcriptome by NGS are illustrated. Depending on the input tissue from which RNA is isolated, a transcriptome of the whole organism or adhesion-related tissue can be generated. See text for details.

A transcriptome represents the entirety of RNA molecules expressed in an organism, a tissue or a certain cell type [12–15] (Figure 2). One has to be aware that this collection includes messenger RNA (mRNA), ribosomal RNA (rRNA), transfer RNA (tRNA), and non-coding RNAs [16–17]. For the identification of adhesion-related genes we are mostly interested in mRNA which comprises only 1–5% of all RNAs produced [18].

For simplicity, in the following sections, we refer to "transcriptome" as the full complement of mRNAs of our target organism, tissue or cell type of interest. We have to keep in mind that the representation of mRNAs in the transcriptome experiment depends on the developmental stage of the organism, its environmental condition, and the selected tissue or body region. Finally, after transcriptome sequencing is completed the researcher usually receives a FASTA file containing the cDNA sequences. A BLAST search facility can be set up (section 2) to search for homologue sequences. The significance of an available transcriptome cannot be overestimated. It can be seen as the cornerstone of downstream applications such as gene isolation, expression studies by ISH (section 3), and functional studies by RNAi (section 4).

#### 1.2 Why transcriptome sequencing?

The identification of proteins involved in the adhesion of an organism will eventually require the isolation of the respective gene. Before next generation sequencing was available, gene isolation proved to be a laborious endeavor. The advent of modern sequencing technologies has changed gene isolation strategies away from approaches, which investigated a single gene at a time, towards an encompassing all-genes-at-once strategy. Therefore, the rationale for performing transcriptome sequencing – commonly referred to as RNA-seq – is based on the relative simplicity, nowadays, of obtaining a substantial collection of transcripts expressed in a specific tissue or an organism [19–23]. Current and future sequencing technologies allow for the generation of the transcriptome of a tissue or an organism of interest with a comparatively low burden on the research budget, and without in-depth bioinformatic expertise on the part of the commissioning researcher. In-house sequencing facilities of universities and institutes as well as commercial service providers will advise on the sequencing strategy. When using current technologies such as Illumina paired-end sequencing, an initial transcriptome will cost no more than a few thousand euros. Such a dataset will provide a reasonable coverage of the transcriptome in question. Depending on the requirement and the research goals, additional data can be produced and added later, e.g., by applying longer-reads strategies, stranded and/or rRNA removed libraries, and libraries of specific tissues.

#### 1.3 Sequencing a transcriptome

With respect to the generation of a transcriptome that contains adhesion related genes of an organism, it can be favorable to only select the tissue that contains the adhesive organs. This can have several advantages: First, it will drastically reduce the complexity of the transcriptome when expressed genes of, e.g., the reproductive organs or the brain are not included. Second, the bioinformatic assembly of such a transcriptome will be facilitated. Third, the costs can be reduced since a higher coverage of bases, i.e., the frequency of how often a base of the transcriptome is sequenced, can more easily be achieved.

For the generation of a transcriptome, the following sequence of steps will usually be necessary (Figure 2): First, total RNA isolation. Sequencing facilities usually prefer to be provided with total RNA of good quality. Current technologies require only small amounts of total RNA (1 µg). Alternatively, tissue flash-frozen in liquid nitrogen and stored at −80 °C could be provided for RNA isolation to be performed at the sequencing facility or the commercial provider. RNA isolation can be straightforward by using, e.g., Trizol or Tri Reagent procedures according to the manufacturers instructions. However, RNA isolation often requires the mechanical disruption and homogenization of the tissue. Total RNA can be stored at −80 °C and shipped on dry ice to the sequencing facility. The following steps are recommended to be performed at the sequencing facility or the commercial provider (see steps 2–8 of Figure 2): After poly (A) selection, RNA is fragmented into pieces of 200–300 bp and reverse transcribed into complementary DNA (cDNA). Next, sequencing adaptors are ligated by using a standard protocol. Alternatively, strand-specific sequencing can be performed [24–25]. Size range selection is performed (about 200 bp) followed by a PCR based amplification step before the library is subjected to next generation (NGS) sequencing. After the raw reads are obtained, bioinformatic data analysis including de-multiplexing, artefact removal and error correction is carried out [12,14]. Finally, the reads are assembled to hypothetical transcripts, which results in the transcriptome of the selected organism, tissue or cell type. This transcriptome consists of the reconstructed transcripts as simple text (FASTA) file format.

#### 1.4 Differential RNA-seq

**Figure 3:**
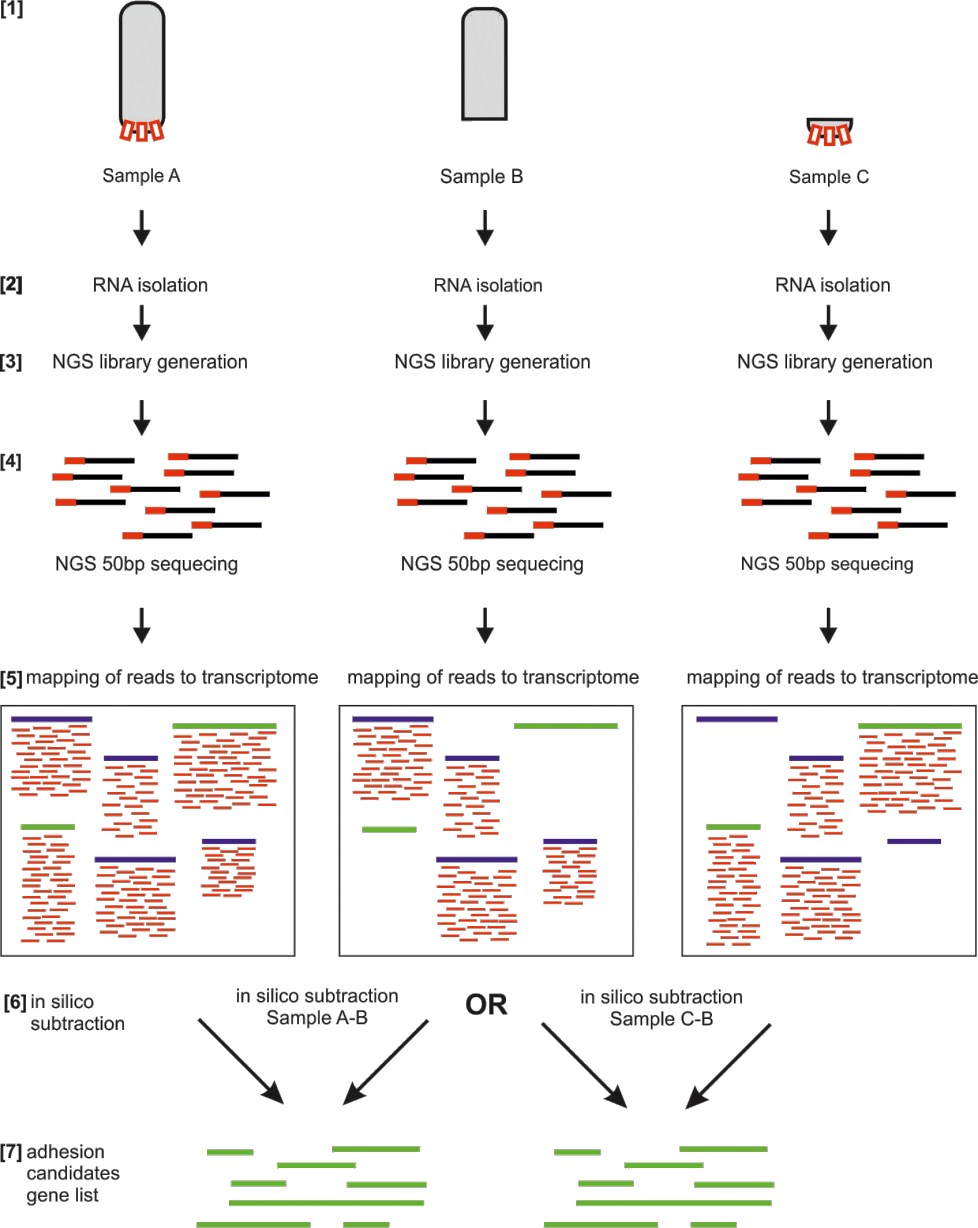
Generation of a differential transcriptome for obtaining a collection of candidate transcript enriched for adhesion-related transcripts. Note that "Sample A" (containing all cells including adhesives cells) minus "Sample B" as well as "Sample C" minus "Sample B" results in an adhesion-enriched candidate transcript list. For small organisms "Sample C" can be difficult to obtain. Therefore, the in silico subtraction of "Sample A" minus "Sample B" is a good option since tissue lacking adhesive organs might be easier to collect. Red rectangles in sample A and C illustrate the adhesive organs in a hypothetical organism. See text for details.

RNA-seq is transcriptome sequencing that reveals a quantitative portrait of mRNAs present within a certain tissue and/or at a certain time point. The basic idea behind differential RNA-seq is the comparison of two conditions to identify the differentially expressed genes [19,23,26–30]. For example, in adhesion research we are interested in the identification of transcripts specifically expressed in the adhesive cells or tissue. Therefore, the experiment needs to be designed in a way that allows the tissue to be obtained, both with and without the cells that produce the adhesive proteins. This can be achieved by amputation, regeneration, collection of different developmental stages, or manipulation of the cellular (for instance by RNAi, see section 4) or physiological conditions. Successful collection of the starting material completely relies on an in-depth knowledge of the morphology of the adhesive organ and the respective organism. A recommended starting point would be RNA isolation of biological triplicates (see ENCODE suggestions for RNA-seq: "Standards, Guidelines and Best Practices for RNA-Seq, The ENCODE Consortium") followed by standard sequencing library generation. In contrast to full transcriptome sequencing where we would aim for long paired-end reads to optimize transcriptome assembly, we would choose cheaper short (50 bp) single reads that would then be mapped to the existing transcriptome (Figure 3). The advantage of this strategy is that one does not need to generate an assembled transcriptome for each replicate of each condition, which would require massive paired-end sequencing and bioinformatic effort. Rather, we generate about 10 million 50 bp single reads of each replicate. Several consecutive steps allow for the identification of differentially expressed transcripts (numbering according to Figure 3): (1) sample preparation, (2) isolation of total RNA, (3) preparation of the NGS library, (4) sequencing of each library, (5) bioinformatic mapping of the reads to the corresponding gene of the transcriptome, (6) bioinformatic subtraction of transcript lists, and (7) generation of the candidate transcript list. Commonly we are faced with the following situation (Figure 3): (A) From the control samples containing all cells of the organism, including the adhesive cells, all reads will be mapped to the transcript present in the transcriptome in a quantitative manner, i.e., transcripts that are highly expressed will be sequenced more often and, therefore, a higher number of mapped reads will be obtained. (B) In samples that lack the adhesive cells the mRNAs of the adhesive proteins will not be represented in the library while all other mRNAs of the machinery of a cell will be present. Therefore, in the sample B, no mapped reads will be obtained for the adhesion-related transcripts whereas all other transcripts are covered with the respective short reads. Finally, the collection of transcripts without mapped reads constitutes the adhesive-transcript candidate list – a highly valuable collection of transcripts for downstream applications. Alternatively, (C) samples containing adhesive cells could be extracted. This sample will generate a library that contains all the mRNAs of the adhesive proteins, boosting the comparisons between samples (Figure 3).

### 2. Creating a local BLAST search facility

#### 2.1 BLAST – basic local alignment search tool

Basic local alignment search tool (BLAST) is a software package to query sequence databases for homologues [31]. Statistical information helps to determine the significance of every alignment. BLAST is widely used to analyze sequencing data and to find candidate genes for further analysis using molecular approaches.

#### 2.2 Establishment of a local BLAST search system

We recommend the software "SequenceServer" (http://www.sequenceserver.com/) to deploy a web-based system to share and query sequence data for similarities [32]. It uses all advantages of recent developments on the NCBI-BLAST+ package [33], is free of charge for academics and has an easy to use web interface.

The setup can be achieved by following the detailed documentation available at the SequenceServer homepage. Briefly, to comply with the requirements a computer or server running a Linux operating system (e.g., Debian GNU Linux; http://www.debian.org/) or MacOS is needed. Besides the NCBI-BLAST+ package, the Ruby scripting language (http://www.ruby-lang.org/) has to be installed. Most Linux distributions perform installation tasks by using a package-management system, e.g., aptitude). SequenceServer setup is performed by the Ruby package management framework rubygems. Further it is needed to define directory-paths to the NCBI-BLAST+ executables and the transcriptome-FASTA-file at the SequenceServer configuration file. SequenceServer is accessible using a web browser immediately after program start, because it uses Ruby’s built in webserver Webrick (http://www.ruby-doc.org/stdlib-2.0/libdoc/webrick/rdoc/WEBrick.html).

Finally, any query sequence such as known adhesion-related transcripts of other organisms, mass spectrometry peptide sequences or candidate transcripts originated from a differential RNA-seq experiment can be compared to the established transcriptome database.

### 3. Spatial gene expression

#### 3.1 Aim of in situ hybridization

**Figure 4:**
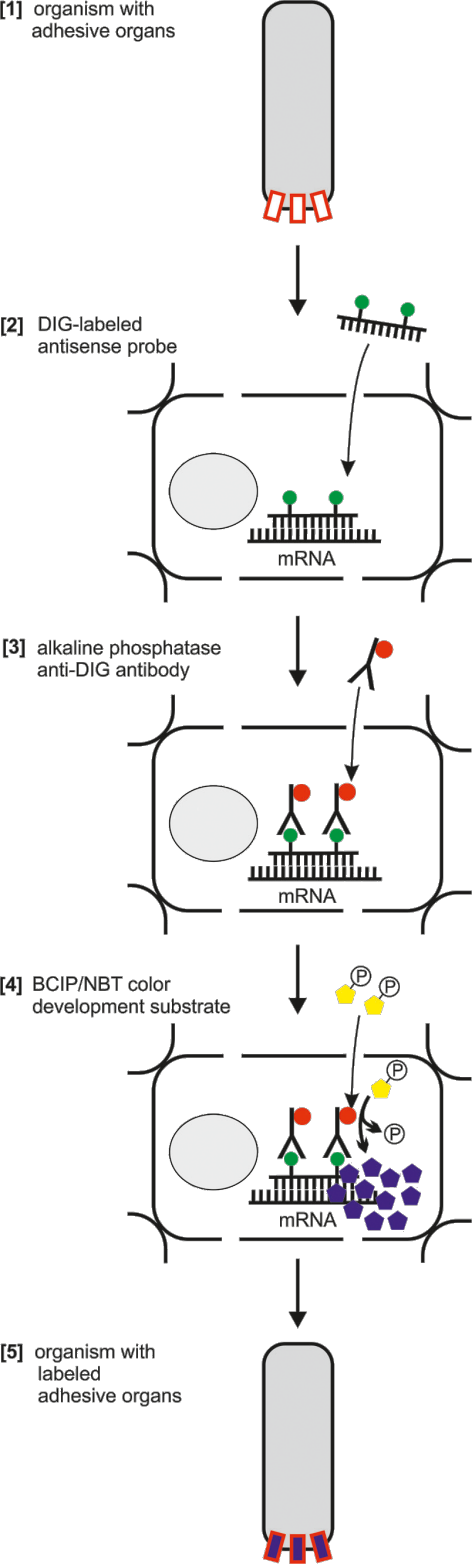
Principle of in situ hybridization. (1) Schematic organism with unstained adhesive organs. (2) The DIG-labelled RNA probe binds to the target mRNA and (3) is detected by an antibody conjugated with a phosphatase. (4) The former colorless substrate becomes dephosphorylated and turns blue. (5) The staining reveals the cells with target gene expression, in this case the adhesive organs.

For detecting the spatial (and temporal) expression of genes within a tissue, ISH is a widespread and straightforward method*.* The principle of ISH can be used to detect various types of nucleic acids [34–37]. In this review we will focus on the visualization of specific transcript expression in the form of mRNA in whole mount specimen and tissue sections. ISH provides a powerful tool to map candidate transcripts from a transcriptome dataset to a distinct tissue or cell type. In bioadhesion research, it can be used to identify and validate gene exclusively expressed in adhesion-related cells, like supportive cells [10] or secretory glands [6,38]. The method described below is based on the complementary binding of digoxigenin labelled nucleotide probes to endogenous mRNA [39] (Figure 4).

#### 3.2 In situ hybridization set-up

Several ways to visualize the probes can be utilized – with fluorescent dyes, with alkaline phosphatase, or horseradish peroxidase reactions. We will present a widely used chromogenic visualization method, based on an alkaline phosphate reaction. The first step is the production of single-stranded RNA probes labelled with digoxigenin (DIG) (Figure 4). Gene specific primer pairs are designed and extended at their 5’ end with a RNA polymerase T7, T3 or SP6 promoter sequence [40]. Regions for ISH probes must be selected carefully and should not have significant similarities to other endogenous transcripts (BLAST search). The size of the probes should range between 500 and 1000 nucleotides. Shorter probes can lead to weak staining results and/or less specificity. cDNA is used as a template for a standard PCR reaction with the gene-specific primers. The purified PCR product serves as a template for in vitro RNA probe synthesis. Depending on which primer (forward or reverse) the polymerase promoter sequence is located, sense or antisense RNA probes are produced. Antisense probes bind to the target mRNA and should lead to a specific ISH pattern, whereas sense probes are often used as a negative control. Purified RNA probes are stable for months at −80 °C.

For most model organisms standardized protocols for ISH are available [41–47]. To successfully stain other organisms, species-specific adaptations may be required. Critical steps are the fixation and the achievement of permeability of the tissue without losing endogenous mRNA or structural tissue integrity. Usually, good results are achieved with a fixation using 4% paraformaldehyde and proteinase K treatment. Treatment times and concentrations vary depending on tissue hardness and size and must be empirically tested for every tissue. If permeability and transparency cannot be achieved in a whole mount specimen, it may be necessary to perform the ISH on tissue sections [48]. After pre-treatments of the tissue, the DIG-labelled RNA probe is added and hybridized to the complementary mRNA (Figure 4). The hybridization products are then detected with an anti-digoxigenin antibody conjugated to alkaline phosphatase. NBT/BCIP (NBT: nitro blue tretrazolium chloride, BCIP: 5-bromo-4-chloro-3-indolyl phosphate) is a colorless substrate that becomes a blue precipitate when it is dephosphorylated. When added to the samples NBT/BCIP leads to a stable blue staining in cells where the anti-digoxigenin antibody is bound (Figure 4). Endogenous phosphatase activity can lead to a false-positive staining. Therefore, it is essential to inhibit phosphatases during the pretreatments of the tissue and to perform valid negative-control experiments.

#### 3.3 Large-scale expression screening

Once the ISH protocol for an organism is adjusted, it provides a powerful tool to perform large-scale expression screens. For example, it might be necessary to study and validate the expression of an adhesion-related candidate transcript list that resulted from previous mass spectrometry or differential gene expression experiments. For high-throughput approaches, in situ robots such as “InsituPro VSi” from Invatis AG are available. For medium scale ISH screenings, a manual 24-well plate system might be useful [10].

The knowledge of spatial and temporal expression patterns is crucial to elucidate the function of genes during development. Therefore, numerous high-throughput in situ screens have been performed in model organisms from developmental biology such as mouse [40,49–52], chicken [43,53–54], zebrafish [55], mekada fish [56–57], the fruit fly *Drosophila* [47,58], the frog *Xenopus* [59–62] and the ascidian *Ciona intestinalis* [63–66]. These screens demonstrate the potential of large-scale ISH for the discovery of new genes. In bioadhesion research, high-throughput expression analyses can be adapted for the identification of genes exclusively expressed in adhesive organs, providing a straightforward method to discover adhesion-related proteins.

### 4. Gene function analyses by RNA interference or TALENs/CRISPR

#### 4.1 What is RNA interference?

**Figure 5:**
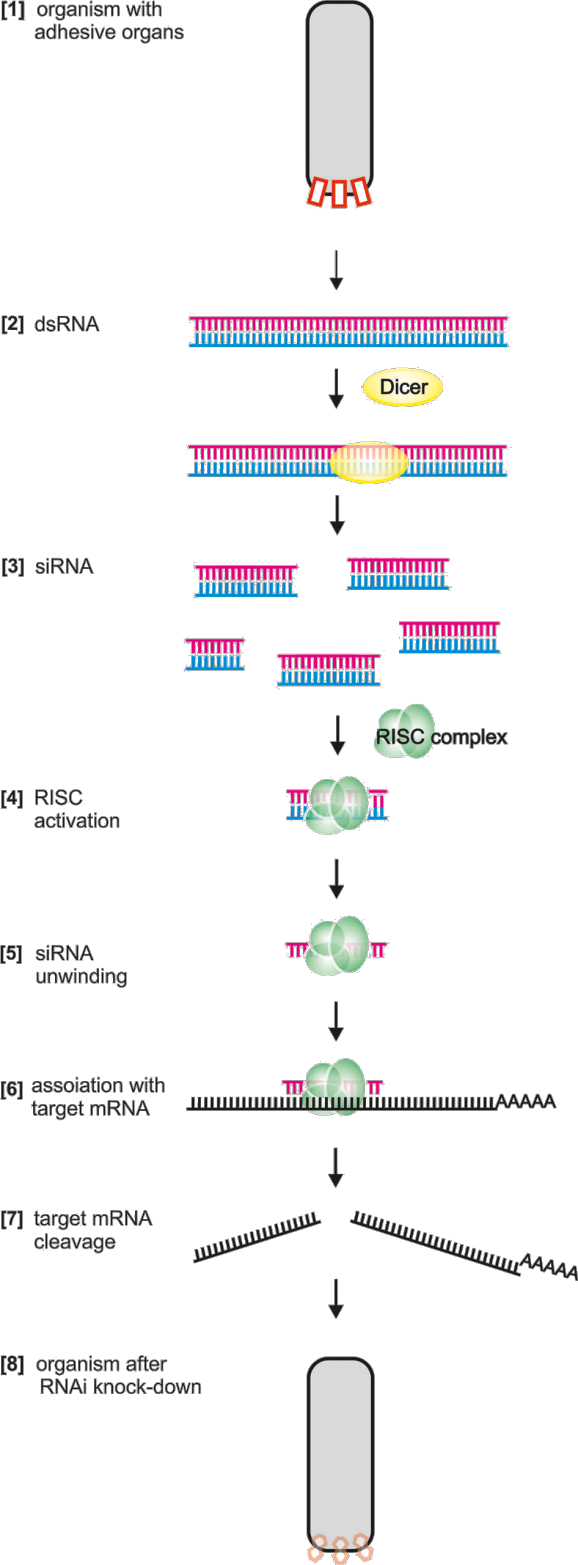
Functional analyses of an adhesion-related gene by RNAi. After the application of dsRNA the mRNA gets degraded. The lack of the corresponding protein affects the function of the adhesive structure and the organism is unable to attach if an essential adhesion-related gene has been targeted. See text for details.

In order to evaluate whether a transcript that is expressed in the adhesive organs of an animal indeed exhibits a role in adhesion, a functional analysis of the gene and its respective protein is necessary. There are several ways to identify the role of a gene, but RNAi offers a fast and direct way. By means of RNAi the mRNA of the gene of interest is broken down and the corresponding protein cannot be produced anymore. The lack of the protein will lead to a deficiency in the function of the cell (Figure 5). In the case of an adhesion-related protein, this could lead to a non-adhesive phenotype [10]. The degradation of the respective mRNA is achieved by the application of a several hundred base pairs long double-stranded RNA (dsRNA) corresponding to the gene sequence or commercially available and bioinformatically designed 20–25 base pairs (bp) small interfering RNAs (siRNAs).

The dsRNA uptake by the cell and gene knock-down results from a complex and multistep mechanism (Figure 5). The exogenous long dsRNA (usually 200–1000 bp in length) is transported to the cell cytoplasm, where it is recognized by a ribonuclease III-like enzyme (Dicer). The Dicer cleaves this long dsRNA in short fragments of 21–22 bp in length. These short fragments are known as siRNAs. Each siRNA is unwound into two single stranded components: The passenger strand, which is degraded, and the guide strand which is recruited by the RNAi-induced silencing complex (RISC). When the guide strand fits to a given complementary mRNA, a protein which makes part of the RISC, known as Argonaute, cleaves the mRNA resulting in its efficient degradation. To date, a standardized RNAi protocol is still not available for some organisms such as the fruit fly [67–68]. This is not the case for the nematode *Caenorhabditis elegans* [69], or planarians [70–72], for which straightforward RNAi protocols are established. The application of RNAi is limited by the efficiency of the uptake of dsRNA, which differs for different genes, organisms and developmental stages. Therefore, preliminary studies are required. RNAi is currently available in a range of different methodologies and is widely used for functional analysis in cellular, animal [73], and genome-wide studies [68,74]. In the context of bioadhesion research, RNAi might also be applied to check if the selected adhesion-related transcripts are actually carrying out the expected function.

#### 4.2 The RNA interference experiment

The first step is the synthesis of the dsRNA. The full-length gene is usually not used for dsRNA synthesis (Figure 5), rather gene-specific sequences between 200 bp to 1000 bp are chosen. Special attention should be paid to the selection of the sequence of the transcript to be knocked-down and highly conserved domains that could also be present in other genes should be avoided. Therefore, for long dsRNA synthesis the sequence identity and uniqueness to the target transcript of the organism needs to be verified [67–68,75–77]. In order to generate the dsRNA, we have frequently used the same primers with which ISH was previously performed. Templates can be generated by standard PCR amplification from cDNA, but this time, with the addition of a RNA polymerase promoter (T7, T3, or SP6) to the 5´ end of both primers (forward and reverse). It is highly recommended to clone and sequence the amplified fragments, however, for a high-throughput screening, the amplified PCR fragments might be used directly and verified only if an interesting phenotype is observed. Following PCR amplification a transcription reaction is performed in two independent reactions to synthesize the two complementary RNA transcripts from the template. Several commercial kits for RNA synthesis are available and can be used according to the manufacturer’s instructions [78–81]. After annealing the RNA strands by in vitro transcription to form the dsRNA, the DNA and single-stranded RNA are removed through a nuclease digestion. After purification, dsRNA is checked for quality and concentration. Finally, aliquots containing the desirable concentrations of dsRNA can be stored at −80 °C or directly used for RNAi experiments.

The dsRNA can be delivered to the target organism by a variety of methods; the most common are soaking, ingestion and injection. In several aquatic organisms like *Hydra*, flatworms, planarians, nematodes, and shrimps, feeding or soaking are the most straightforward methodologies for delivering dsRNA. The organisms have to be immersed in a medium containing dsRNA. Another strategy is ingestion, by inducing target organisms to feed on other organisms like bacteria expressing the desirable dsRNA [69,82–84], or transgenic plants for feeding insects [85]. Also the combination of methods like the enrichment of natural diets, for example, liver paste and *Artemia* enriched with engineered bacteria to feed planarians and *Hydra* [72,86]. Lastly, microinjection has been applied in several species, like the harvestmen Opiliones [87] and tardigrades [88]. The suitability of each delivery method depends on the organism being studied. Experimental animals should be incubated or injected with dsRNA solution for an appropriate period of time. The incubation time is extremely variable and is dependent on cell turnover in the target tissue. Gene knock-down in biological adhesion has been achieved by using in vitro designed long dsRNA in the flatworm *Macrostomum lignano* [10].

Importantly, control experiments should include an RNAi molecule against a heterologous sequence absent from the genome of the target organism. For example, in the flatworm *M. lignano* a dsRNA of the firefly luciferase sequence of 1002 bp was used as negative control [78,80–81,89]. Regarding the validation of experiments, quantitative real-time PCR is the most straightforward way to direct evaluate if the mRNA was in fact knocked down. Also, ISH against the target mRNA could provide a representation of the results when comparing treated samples and controls, albeit not in a quantitative manner.

#### 4.3 New approaches: TALENs and CRISPR

Genome editing technologies offer a potential tool for bioadhesion research. The central idea is to specifically mutate the genomic region of the gene of interest to inhibit the production of functional mRNA and protein. While RNAi experiments are a robust and useful tool, the results of these experiments are temporary, preventing longer-term evaluations. Traditionally, zincfinger nucleases have been used for genome editing [90] but they have limitations in the freedom to select a particular genomic region, and they are expensive. Recently, two customised genome editors have become available and these have gained acceptance from the scientific community. First, the transcription activator like effector nucleases (TALENs) [91–92], cause the fusion of DNA binding domains derived from TALE proteins with the *Fok*l restriction endonuclease. Basically, TALENs induce DNA double-stranded breaks that stimulate the cellular DNA repair mechanisms enabling custom modifications [91,93]. The second genome editor is the clustered regulatory interspaced short palindromic repeat (CRISPR/Cas) [94–97], which uses a guide RNA and a protein called Cas9 endonuclease to enable a sequence-specific cleavage of homologous target double-stranded DNA. Both TALENs and CRISPR/cas genome-editing tools allow for gene knock-out, knock-in (when a desired gene is inserted) or the modification of genes, and represent a powerful method capable of providing conclusive information for evaluating gene function. However, these technologies require genomic information of the target organism or the gene of interest. Also, the microinjection delivering system in single-cell embryos are compulsory for these technologies. Nevertheless, TALENs and CRISPR appear to work in principle in most organisms and might be a useful tool to study gene function in diverse organisms.

## Conclusion

The identification of adhesion-related genes and proteins is a challenging task. Certain organisms allow the collection of the glue and direct analyses by mass spectrometry or biochemistry. Small organisms can exhibit remarkable adhesive performance but their tiny size impedes the direct collection of the glue. Therefore, other approaches are necessary for identifying adhesive molecules. A molecular biological approach provides the means to identify adhesion-related transcripts in these organisms and allows their expression and function to be studied. Nowadays, even a small research group can use high-throughput sequencing platforms to generate a transcriptome of an organism. Differential gene expression can be highly useful to narrow down the number of candidate transcripts. In order to further confirm the expression of genes of the candidate list – which can also be derived from a mass spectrometry experiment – ISH needs to be employed. Next, the possible role of an adhesion-candidate transcript can be studied by adapting gene knock-down using RNAi or gene knock-out by TALENs or CRISPR for the respective organism. The need for new strategies in adhesion research demands efforts in key molecular biology technologies. Enhancing our ability to understand in vivo adhesive molecules is essential for exploring biomimetic approaches to synthesising new adhesive products. A molecular biology approach can help to facilitate the search for new adhesives across the animal phyla.
